# Innocuous pressure sensation requires A-type afferents but not functional ΡΙΕΖΟ2 channels in humans

**DOI:** 10.1038/s41467-021-20939-5

**Published:** 2021-01-28

**Authors:** Laura K. Case, Jaquette Liljencrantz, Nicholas Madian, Aaron Necaise, Justin Tubbs, Micaela McCall, Megan L. Bradson, Marcin Szczot, Mark H. Pitcher, Nima Ghitani, Eleni Frangos, Jonathan Cole, Diana Bharucha-Goebel, Dimah Saade, Tracy Ogata, Sandra Donkervoort, A. Reghan Foley, Carsten G. Bönnemann, Håkan Olausson, M. Catherine Bushnell, Alexander T. Chesler

**Affiliations:** 1 National Center for Complementary and Integrative Health, NIH, Bethesda, MD USA; 2 Department of Anesthesiology, University of California, San Diego, CA USA; 3 Department of Anesthesiology and Intensive Care, Institute of Clinical Sciences, Sahlgrenska Academy at University of Gothenburg, Gothenburg, Sweden; 4 Centre of Postgraduate Medical Research and Education, Bournemouth University, Bournemouth, UK; 5 National Institute of Neurological Disorders and Stroke, National Institutes of Health, Bethesda, MD 20892 USA; 6 Center for Social and Affective Neuroscience, Department of Clinical and Experimental Medicine, Linköping University, Linköping, Sweden

**Keywords:** Peripheral nervous system, Somatosensory system

## Abstract

The sensation of pressure allows us to feel sustained compression and body strain. While our understanding of cutaneous touch has grown significantly in recent years, how deep tissue sensations are detected remains less clear. Here, we use quantitative sensory evaluations of patients with rare sensory disorders, as well as nerve blocks in typical individuals, to probe the neural and genetic mechanisms for detecting non-painful pressure. We show that the ability to perceive innocuous pressures is lost when myelinated fiber function is experimentally blocked in healthy volunteers and that two patients lacking Aβ fibers are strikingly unable to feel innocuous pressures at all. We find that seven individuals with inherited mutations in the mechanoreceptor *PIEZO2* gene, who have major deficits in touch and proprioception, are nearly as good at sensing pressure as healthy control subjects. Together, these data support a role for Aβ afferents in pressure sensation and suggest the existence of an unknown molecular pathway for its detection.

## Abstract (web summary)

The mechanisms underlying deep pressure sensing are not fully understood. Here the authors demonstrate that while two individuals lacking Aβ fibers demonstrate impaired deep pressure sensing, seven individuals with PIEZO2 loss of function mutations display normal deep pressure responses.

## Introduction

Interoceptive signals from deep tissues are powerful modulators of our physiological state and serve as important indicators of potential tissue damage, inflammation or disease^1^. Sensory innervation of our muscles, joints and connective tissues provide a wealth of conscious and unconscious information about the state of our bodies when at rest or in motion^2^. Additionally, pressure sensation is a major component of our affective interactions, from the pleasure and comfort associated with hugs to the health benefits of stretching and massage therapy. While our mechanistic understanding of touch perception has grown significantly in recent years, how deep tissue sensations are detected and encoded remain less clear.

The somatosensory system detects both external and internal stimuli. Afferent projections of primary sensory neurons innervate sites throughout the skin and body where they are activated by a wide range of physical and chemical stimuli. The diverse nature of these stimuli is reflected by the heterogeneity of sensory neuron subtypes, their anatomical specializations, physiological properties, and the receptor molecules they express. For example, gentle cutaneous touch sensation is mediated by several types of mechanoreceptors, each having unique ending types, molecular profiles, and physiological properties^3^. Generally speaking, thickly myelinated Aα and Aβ fibers are fast conducting and critically important for proprioception and touch, respectively, whereas the thinly myelinated Aδ and unmyelinated C fibers conduct more slowly and play prominent roles in thermosensation, chemosensation, and nociception^4^. There are notable exceptions to this generalization; for example, a prominent subtype of C fibers is activated by gentle stroking stimuli^5^ and a subset of Aβ fibers have been recently characterized that respond only to painful mechanical stimuli^6^.

The majority of our understanding of peripheral somatosensory neurons comes from studying the afferents that target skin. What is known about non-cutaneous mechanosensation? Most research on interoception focuses on the vagal system. Interest in visceral sensation occurring via dorsal root ganglion neurons has largely centered on pain in relation to disorders such as Irritable Bowel Syndrome or Crohn’s Disease. Similarly, investigations of muscle sensation have mainly focused on either Aα-type proprioceptors that are required for tracking body position^2^ or on the Aδ and C-type nociceptors that signal muscle pain^7,8^. Far less is known about non-painful pressure sensations from muscle, fascia, and deep tissues, despite the unique purposes they clearly serve. Intriguingly, some evidence exists suggesting that both myelinated and unmyelinated sensory afferents innervating muscles can respond to innocuous muscle pressures^9–12^. Nevertheless, the contributions of specific types of afferents to deep tissue sensations remain unknown.

Here, we investigated three aspects of pressure sensation in humans. First, we asked which sensory neuron fiber types mediate our ability to perceive deep pressure on the legs and hands. Second, we investigate whether this type of innocuous pressure stimulus engages the same molecular transduction pathway- ΡΙΕΖΟ2- as gentle touch sensation and proprioception^13–15^. Third, we developed an assay to assess the roles of gentle touch and deep tissue sensation in an active behavioral task. For our studies, we worked with healthy adult volunteers and two cohorts of patients with rare conditions affecting their somatosensory primary afferent systems.

Our first patient cohort consisted of two sensory neuronopathy individuals with selective loss of myelinated Aβ type sensory fibers after a rare virus or autoimmune induced neuronopathy syndrome^16–18^. These patients have been studied extensively (e.g.,^19^) and have profound discriminative touch and proprioception deficits^20,21^. Despite these profound sensory deficits, both individuals perceive cutaneous temperature and pain and have affective responses to skin stroking, where unmyelinated afferents are known to be critical^5^. The phenotypes of the Aβ-deafferented individuals bear striking similarity to our second patient cohort, individuals with inherited bi-allelic loss-of-function mutations in the mechanically-gated ion channel ΡΙΕΖΟ2 (ΡΙΕΖΟ2 Deficiency Syndrome). ΡΙΕΖΟ2 is an essential receptor for mechanical stimuli in multiple species^15,22,23^ that is required for normal proprioception, vibration sensing and touch discrimination^13–15^. Individuals with ΡΙΕΖΟ2 Deficiency Syndrome also exhibit neonatal hypotonia, hip dysplasia, joint hypermobility and contractures, progressive scoliosis, delayed acquisition of motor milestones in the absence of muscle weakness, and deficits in interoceptive sensations such as from the mouth, stomach, and bladder^13,24,25^.

Our findings demonstrate that, as for touch and proprioception, Aβ fibers are required for pressure sensation. However, unlike several other types of non-painful mechanosensation, pressure sensing does not require ΡΙΕΖΟ2 function. Together these findings offer insight into the neural and molecular pathways for pressure sensation in humans.

## Results

### Pressure sensation requires myelinated A-fiber function

**Fig. 1:**
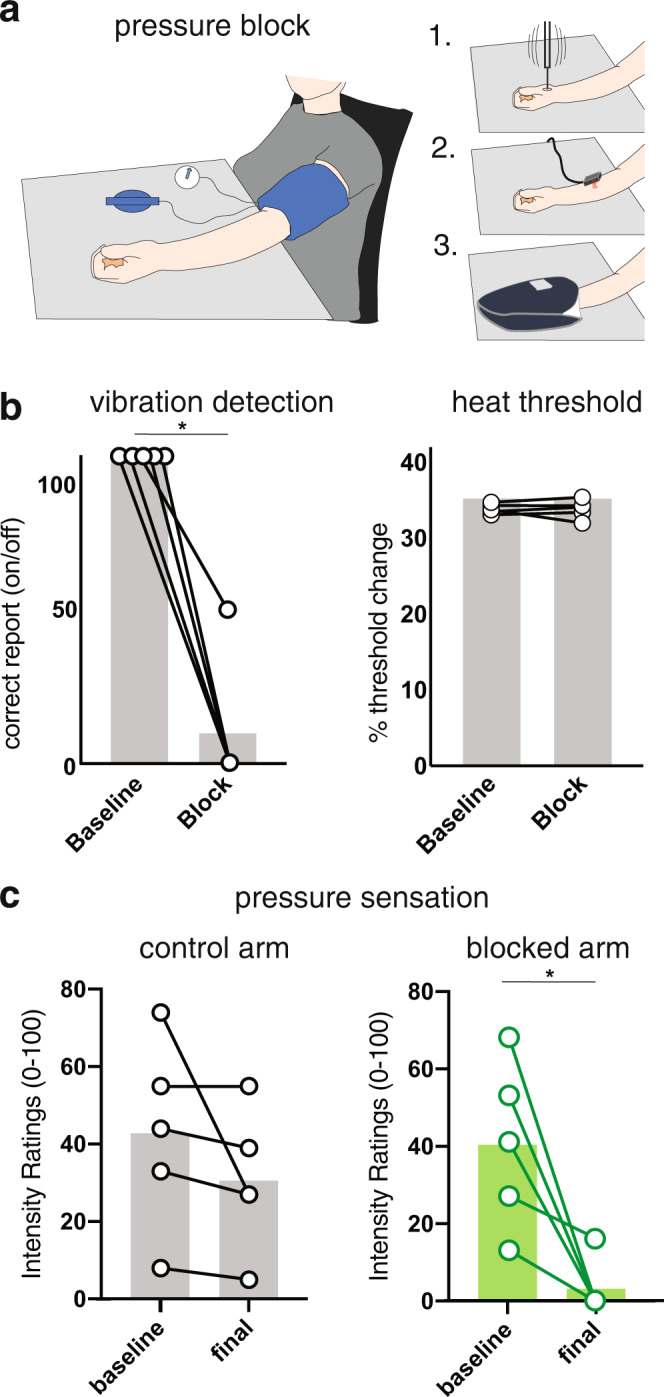
Aβ-fiber blockade inhibits innocuous pressure sensation in the hand. **a** A cartoon depicting the administration of a pressure block during quantitative sensory evaluation. A blood pressure cuff was placed on the upper arm of five healthy adult volunteers and inflated to ~100 mmHg above the participant’s systolic blood pressure. Repeated tests of vibration (1), heat threshold (2), and deep pressure (3) perception were conducted both before and after the placement of the cuff. A barrier blocked visual perception of the test stimuli and noise-isolating headphones played white noise to mask auditory cues. **b** Vibration sensation (left graph), which is known to be mediated by Aβ afferents, was observed to be substantively eliminated during the course of the nerve block before the heat detection threshold (right graph) noticeably changed. Participants were asked to report when the vibrating probe turned on/off; correct scores indicated preserved sensation. At baseline all participants performed at 100%; a large drop in performance of 50–100% was observed in all participants before pressure testing was conducted (one-sided paired permutation test *p = 0.03). Heat thresholds (right graph) were determined using a thermode placed on the ventral forearm. Increased temperature threshold indicates decreased sensitivity. Heat thresholds were unaltered at the time of pressure testing (one-sided paired permutation test *p* = 0.47). *N* = 5 healthy participants. **c** Pressure sensing with the hand massage device. Pressure ratings between the left and right arms did not differ at baseline (one-sided paired permutation test *p* = 0.16). At the time of pressure testing, after loss of vibration, intensity ratings were lower on the blocked arm than on the control arm (one-sided paired permutation test *p = 0.03). Ratings dropped significantly more for the blocked arm than the control arm (one-sided paired permutation test *p = 0.03). *N* = 5 healthy participants.

Which fiber types mediate the sensation of innocuous or pleasant types of deep pressure? We sought a controlled and simple way to dissociate the contributions of myelinated A fibers from unmyelinated C fibers during quantitative sensory evaluations of healthy participants. Blood pressure sleeves are commonly used in the clinic to produce nerve blocks as a means to assess sensory nerve function (“pressure block”; Fig. 1a). Importantly, as this type of nerve block develops, the larger myelinated A fibers stop functioning well before the smaller unmyelinated C fibers do^26–28^. We first confirmed that nerve blocks caused by blood pressure cuffs applied to the upper arm led to participants reliably losing cutaneous vibration sensation (known to be mediated by Aβ fibers) on their hands before heat detection by C fibers was affected (*N* = 5; Fig. 1b). We next asked how the perception of controlled and oscillating gentle squeezing of the hand by a commercial hand massager device (see Methods) changed at the point in the nerve block when vibration sensation was gone but heat perception was unaltered. Participants were asked to rate the intensity of the pressure stimuli on a scale from no sensation to extremely intense (0-100). Ratings of pressure sensation from the control, unblocked arm remained stable throughout the testing session (Fig. 1c). By contrast, just as we saw for vibration detection, the ability to sense this type of pressure was completely lost even though heat perception was unchanged (Fig. 1b).

### Neuronopathy patients who lost Aβ fibers cannot detect non-painful pressures

We wondered if the two previously studied Aβ-deafferented patients would be able to sense deep pressure since this modality had not previously been assessed. Therefore, we invited both to visit so we could directly compare their performance on a series of quantitative sensory tests with those of age and gender-matched healthy volunteers.

**Fig. 2:**
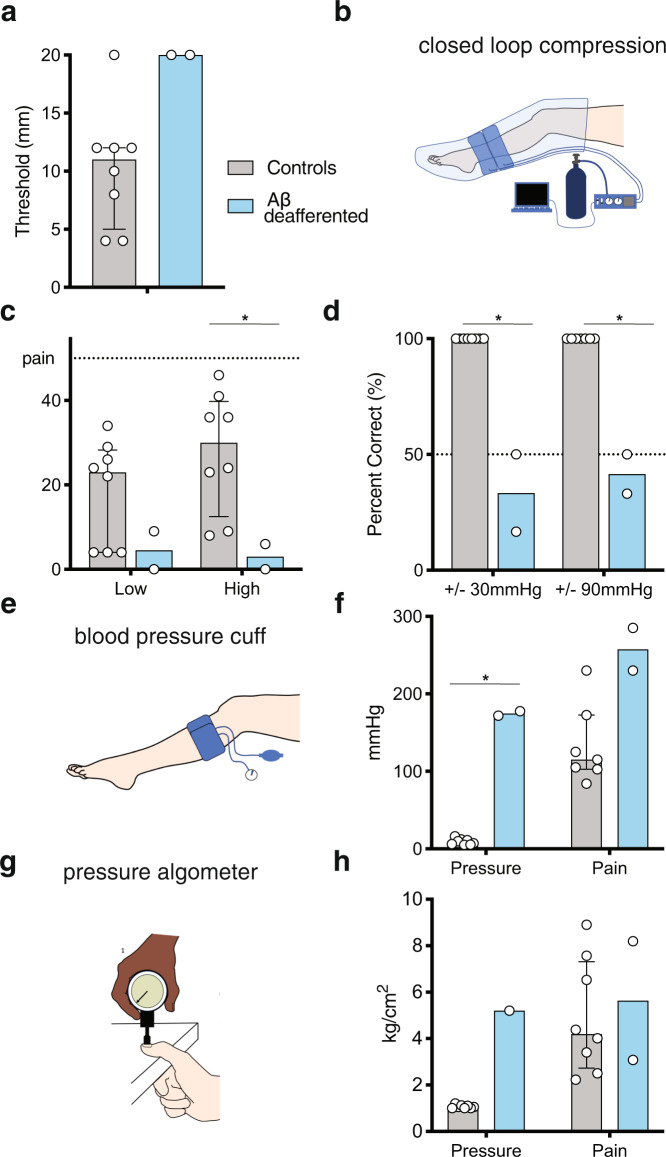
Individuals lacking Aβ-afferents have impaired pressure sensation. **a** Two-point discrimination task on palm. Aβ-deafferented participants (*N* = 2) were unable to discriminate ≤20 mm cutoff. 7/8 control participants performed near the normal range^49^ (one-sided permutation test *p* = 0.07). **b** A cartoon depicting the custom-built leg compression sleeve device (from Case et al.^50^). A computer controlled inflation rate and pressure around the left calf. **c**. Intensity of the compression sleeve oscillating between 10–30 mmHg (low) or 15–65 mmHg (high) was rated on a VAS scale (no sensation = 0; pain threshold = 50). Controls (*N* = 8) rated significantly higher intensity than patients (*N* = 2) for high (one-sided permutation test *p = 0.02) but not low (one-sided permutation test *p* = 0.16) pressure. Bars display median ratings and interquartile intervals. **d** Two-alternative forced choice discrimination task of sequential pressure pulses differing by 30 mmHg. For <75% accuracy, differences were increased to 60 and 90 mmHg. Aβ-deafferented participants (*N* = 2) were significantly poorer than controls (*N* = 8) at 30 mmHg differences (chance = 50%; one-sided permutation test *p = 0.02) and remained unable to discriminate 90 mmHg differences. Bars display median ratings and interquartile intervals. **e** Cartoon depicting the standard manual blood pressure cuff used to obtain pressure thresholds. **f** A blood pressure cuff (**e**) was inflated on the calf until first report of pressure sensation, then pain. Both thresholds were elevated in Aβ-deafferented participants (*N* = 2) compared to controls (*N* = 8) (pressure threshold one-sided permutation test * *p* = 0.02; pressure pain threshold one-sided permutation test *p* = 0.06). Bars display mean ratings and SD. **g** To examine pressure perception over a smaller surface area, a pressure algometer was pressed on the left thumbnail. **h** The experimenter gradually increased algometer pressure from 0 kg/cm^2^. Participants reported first perception of pressure (pressure threshold) or pain (pressure pain threshold). The pressure detection threshold was higher in Aβ-deafferented participants (*N* = 1) than in healthy controls (*N* = 8) (one-sided permutation test *p* = 0.11), but pressure pain thresholds did not differ significantly (*N* = 2 patients and 8 controls) (one-sided permutation test *p* = 0.38). Bars display median ratings and interquartile intervals.

As reported previously, both Aβ-deafferented patients performed very poorly on a two-point discrimination task performed on the thenar eminence of the palm (Fig. 2a), consistent with findings that these individuals lack conscious touch sensation^5,29^. To quantify their sensitivity to pressure, we designed a custom leg sleeve that could be inflated to maintain specific forces via a closed loop sensor and developed rating assays that probed several aspects of pressure sensation (Fig. 2b). Each participant was asked to rate the intensity of a slowly oscillating pressure stimulus (oscillating between 15 and 65 mmHg) on a linear scale ranging from no sensation (0) to the first sensation of pain (threshold; 50) to a pain they would not be willing to experience again that day (tolerance; 100) (Fig. 2c). Age and gender-matched healthy volunteers readily reported these changes in pressure and described the sensation as moderately intense but non-painful. By contrast, both Aβ-deafferented patients were completely unable to detect the oscillating pressure, rating the intensity at or near 0 for each trial. Furthermore, the Aβ-deafferented participants were equally incapable of perceiving a lower pressure oscillating stimulus (10–30 mmHg), a task all control participants reported perceiving (Fig. 2c).

A previous study found that the Aβ-deafferented participants could unconsciously detect slow and gentle brushing stimuli in a two-alternative forced choice discrimination task^5^. We wondered whether the same might be true for deep pressure sensation. Participants were asked to discriminate between different intensities of pressure in a forced choice assay, even if they could not consciously perceive a stimulation. Healthy controls were able to distinguish between two stimuli that differed by as little as 30 mmHg of pressure with perfect accuracy (100%). Aβ-deafferented patients were completely unable to discern differences of even 60 or 90 mmHg (Fig. 2d), suggesting that information about non-noxious deep pressure cannot be sensed at either a conscious or unconscious level by these individuals.

Nociceptive information is carried by distinct sensory afferents from those that detect innocuous stimuli. To determine whether Aβ-deafferented patients could detect higher intensity deep pressure stimuli that is noxious, we used a smaller pressure cuff that could generate greater forces (Fig. 2e). For control participants, the pressure detection threshold measured with this smaller cuff was between 10 and 20 mmHg, whereas the pressure pain threshold was between 100 and 250 mmHg (Fig. 2f). By contrast, the Aβ-deafferented patients required more than ten times the pressure detected by controls before they could detect the cuff inflation. In fact, the patients’ detection threshold was quite similar to the pain threshold for healthy participants, suggesting that they utilized nociception to detect pressure (Fig. 2f). Consistent with this hypothesis, the patients’ thresholds for pain were not significantly different from those reported by controls, indicating that pressure pain perception is preserved in these individuals (Fig. 2f). Furthermore, testing a completely different type of pressure—controlled pressure applied to the thumbnail—produced similar results: significantly elevated thresholds for pressure perception in patients, but similar thresholds for pressure pain. Consistent with our findings from nerve blocks in healthy volunteers, data from these two neuronopathy patients fully support the conclusion that Aβ afferents are required for the detection of innocuous pressure but not for detection of painful pressure (Fig. 2 g, h).

### The mechanoreceptor ΡΙΕΖΟ2 is not required for pressure sensation

**Fig. 3:**
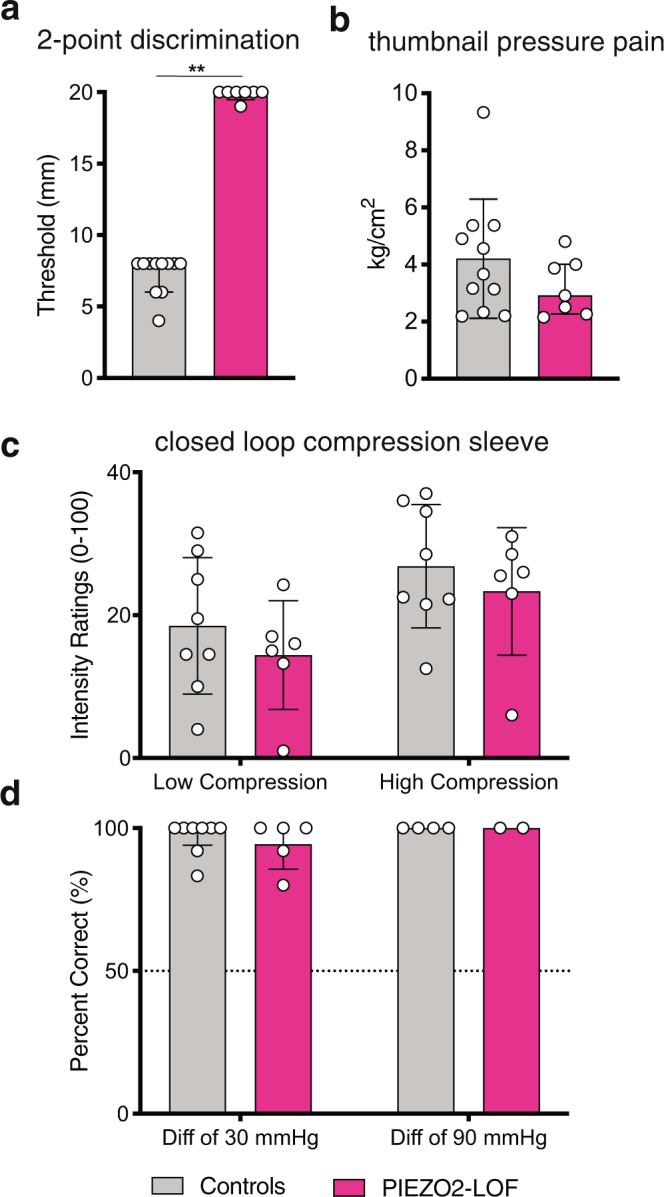
ΡΙΕΖΟ2 is not required for pressure sensation. **a** Two-point discrimination task performed on the thenar eminence of the palm (glabrous skin). Participants with ΡΙΕΖΟ2 Deficiency Syndrome (ΡΙΕΖΟ2 LOF; *N* = 7) were unable to perform the test at or below cutoff of 20 mm whereas age- and gender-matched controls (*N* = 11) had normal discrimination limits (one-sided permutation test **p ≤ 0.001). Bars display median ratings and interquartile intervals. **b** Pressure algometer task as described in Fig. 2h legend. There was no difference between ΡΙΕΖΟ2 LOF participants (*N* = 7) and controls (*N* = 13) (one-sided permutation test *p* = 0.13). Bars display median ratings and interquartile intervals. **c** Intensity of the compression sleeve oscillating between 10 and 30 mmHg (low) or 15 and 65 mmHg (high) was rated on a VAS scale (no sensation = 0; pain threshold = 50). Both ΡΙΕΖΟ2 LOF participants (*N* = 6) and healthy controls (*N* = 8) identified changes in pressure as obvious but innocuous. Patient ratings did not differ significantly from controls (low pressure one-sided permutation test *p* = 0.45; high pressure one-sided permutation test *p* = 0.68). Bars display median ratings and interquartile intervals. **d** Two-alternative forced choice discrimination task between pressure pulses differing by 30 or 90 mmHg using the leg compression sleeve (Fig. 2b). Both ΡΙΕΖΟ2 LOF participants (*N* = 5) and healthy controls (*N* = 8) were able to distinguish differences of 30 mmHg with nearly 100% accuracy (one-sided permutation test *p* = 0.69). One patient was omitted from the 30 mmHg task due to experimenter error, but detected a 15 mmHg difference above chance. *N* = 2 patients and *N* = 4 controls were also tested on 90 mmHg and all displayed 100% accuracy. Bars display median ratings and interquartile intervals.

The finding that the Aβ-deafferented patients are unable to feel innocuous pressure raised the intriguing possibility that ΡΙΕΖΟ2 might also be required for this type of mechanosensation. Consistent with such a hypothesis, *PIEZO2* is abundantly expressed in nearly all large diameter and presumptive A-type neurons in mice^30^. However, a role for this molecule in sensing innocuous pressure has not been quantitatively evaluated. We therefore tested the performance of 7 patients with ΡΙΕΖΟ2 Deficiency Syndrome, 4 previously reported^31^ and 3 newly identified, in the closed loop pressure cuff assay relative to their own age and gender-matched controls. Patients were unable to perform the two-point discrimination task up to 20 mm (Fig. 3a) and had normal pressure pain thresholds (Fig. 3b), consistent with our previous findings^16,22^. To our surprise, however, the ΡΙΕΖΟ2 Deficiency Syndrome patients rated the perceived intensity of the oscillating pressure stimuli on the leg similarly to control participants (Fig. 3c). Notably, ΡΙΕΖΟ2 Deficiency Syndrome patients were able to detect the smallest pressure differential tested (30 mmHg) with near perfect accuracy (Fig. 3d), a level of performance indistinguishable from controls. This contrasts with the greatly increased thresholds for detection of light touch from von Frey filaments we previously reported in ΡΙΕΖΟ2 Deficiency Syndrome patients^31^. Together, these data unexpectedly reveal that ΡΙΕΖΟ2 is not required for non-noxious deep tissue pressure sensation.

### A-fibers but not ΡΙΕΖΟ2 are required in an active pressure task

**Fig. 4:**
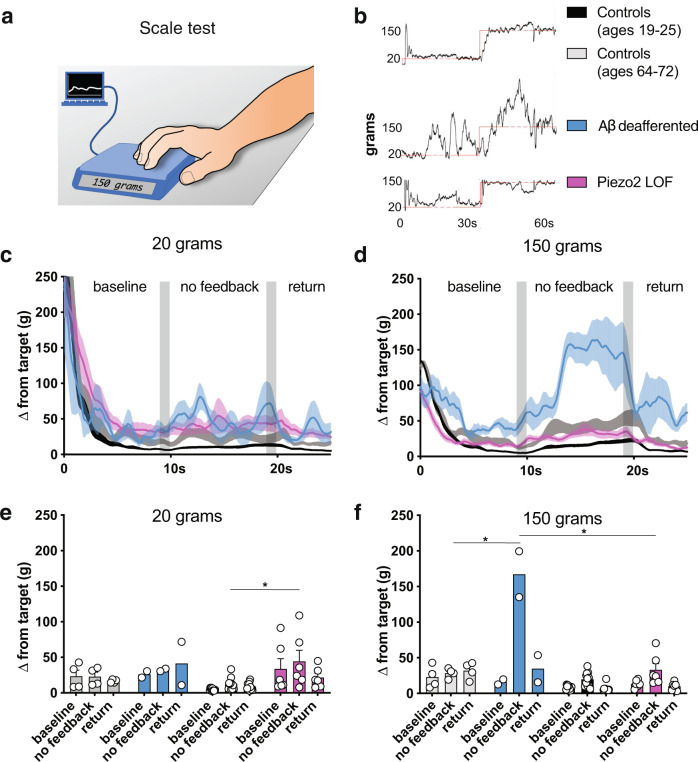
Deficits in pressure sensation impair performance in an active mechanosensation task. **a** Cartoon depicting the digital scale developed to quantify active pressure sensing. Participants applied pressure with their fingertips to match a target on the computer screen. **b** Example force trace from a control participant. Participants attempted to match their force readout (black) to the target (red) at 20 g (cutaneous) and 150 g (putative deeper pressure). After 15 s the line disappeared (dotted red lines) and participants were instructed to maintain their applied force for 10 s (no feedback; gray bar). Afterwards, the line reappeared before moving to the next value. **c** Mean deviation from target value over 1 s at 20 g in controls (gray and black traces, *N* = 17), Aβ-deafferented patients, (blue, *N* = 2) and ΡΙΕΖΟ2 LOF (magenta, *N* = 6). Bars display standard error. **d** Mean deviation from target value over 1 s at 150 g. ΡΙΕΖΟ2 LOF patients (*N* = 6) did not significantly differ in maintaining 20 versus 150 g without visual feedback (one-sided permutation test *p* = 0.70); younger healthy controls (*N* = 11) were poorer at 150 g (trend; one-sided permutation test *p* = 0.06). Aβ-deafferented participants (*N* = 2) appeared worse at 150 g without visual feedback, though this difference was not significant (one-sided permutation test *p* = 0.24); older controls (*N* = 6) were poorer at 150 g (one-sided permutation test *p* = 0.046). Bars display standard error. **e** Alternate representation of target deviation at 20 g (average error in final 5 s of each interval; + = visual feedback and − = no feedback). Both Aβ-deafferented (*N* = 2; blue) and ΡΙΕΖΟ2 LOF (*N* = 6; magenta) participants had more difficulty without visual feedback than older (*N* = 6) and younger (*N* = 11) healthy controls (one-sided permutation tests *p* = 0.18 and *p = 0.007, respectively). Bars display standard error. **f** Alternate representation of target deviation at 150 g. Both Aβ-deafferented (*N* = 2) and ΡΙΕΖΟ2 LOF (*N* = 6) participants had more difficulty than older (*N* = 6) or younger (*N* = 11) controls (one-sided permutation test *p = 0.03 and *p* = 0.06, respectively), and Aβ-deafferented patients had greater difficulty than ΡΙΕΖΟ2 LOF participants (one-sided permutation test *p = 0.03). Bars display standard error.

Many daily activities involve pressure sensation, from the ability to detect the pressure of sitting to the feedback associated with pressing a touch screen. Performance of such tasks involves integration across multiple sensory modalities (e.g., texture detection, temperature sensation, and visual input). We therefore developed an assay to assess the contribution of pressure detection in a task requiring multi-sensory feedback (Fig. 4). Participants were asked to press on a digital scale while receiving visual feedback on a computer screen (Fig. 4a, see Methods). They performed a simple ‘match to sample’ task in which they applied pressure with their fingertips to float a line to a target pressure. After a defined period of 15 s, the screen went blank and the participant was tasked with maintaining the same force in the absence of external (visual) feedback until the target feedback reappeared 10 s later (Fig. 4b). Traces from each of these trials were analyzed for how many grams the applied pressure deviated from the target (Fig. 4c). Based on pilot studies, we chose to compare performance on a test that primarily measured cutaneous touch acuity (20 g) to a test we predicted would evoke deeper tissue sensation (150 g). At the start of each trial, all participants quickly narrowed in on the target within ~5 s (settling time). This was followed by a period of stabilization. Healthy volunteers were able to maintain ~20 g of force without visual feedback (though not quite as well as was possible with visual feedback) (Fig. 4c, e). Both the Aβ-deafferented and the ΡΙΕΖΟ2 Deficiency Syndrome groups had slightly more difficulty with this task, consistent with their deficits in gentle touch sensation (Fig. 4c, e). Once the target force increased, the performance of the two patient groups diverged significantly. Maintaining 150 g without visual feedback was slightly more difficult than 20 g for the control participants and significantly harder for ΡΙΕΖΟ2 Deficiency Syndrome patients, who have the ability to sense pressure (Fig. 4d, f). In contrast, the Aβ-deafferented patients were completely unable to maintain the 150 g of pressure without visual feedback, showing much greater difficulty than either the controls or ΡΙΕΖΟ2 Deficiency Syndrome patients (Fig. 4d, f). Together these results demonstrate that Aβ neurons are essential for active pressure sensing and that ΡΙΕΖΟ2 is not required for this type of mechano-sensing task.

## Discussion

How non-painful pressure is detected and perceived has remained unresolved. Previous studies in humans using anesthetic or pressure blocks have suggested that cutaneous sensation plays a minimal role^26,27^, indicating the existence of afferents dedicated to sensation from deep tissues. For example, a study performing cutaneous anesthesia eliminated light touch sensation by von Frey filaments except for the single strongest von Frey fiber, which was described by participants as a sensation of “deep pressure”^27^. Consistent with this view, our study offers insights into pressure sensation by evaluating two groups of human patients with different rare mechanosensory deficits, in addition to healthy individuals undergoing a pressure block.

The detection of light touch on the skin by the slowly adapting Merkel cell-neurite complex and the importance of ΡΙΕΖΟ2 in this type of response has been established^31,32^. Similarly, both Aβ-deafferented and ΡΙΕΖΟ2 Deficiency Syndrome patients are significantly impaired in sensing light touch and have difficulties actively maintaining light pressures with their fingertips. In contrast, we found significant differences when we probed deeper pressures; ΡΙΕΖΟ2 Deficiency Syndrome patients perceive deep pressure normally whereas Aβ-deafferented patients and healthy individuals with transient Aβ deactivation cannot. Thus, similar to touch, these findings support the idea that a specialized set of myelinated sensory afferents mediate innocuous pressure sensation whereas painful pressures are detected by thinly myelinated or unmyelinated fibers.

What are the fibers mediating pressure sensing? One possibility is that cutaneous myelinated mechanoreceptors with higher activation thresholds can signal pressure but without causing pain. This could be the case with the ΡΙΕΖΟ2-deficient individuals who retain the ability to detect von Frey filaments at the upper range of forces. Such an explanation is consistent with the existence of unknown molecular pathways for detecting higher forces in cutaneous sensory afferents. Additionally, our results favor that deep pressure sensation also arises from distinct and dedicated types of peripheral neurons. Somatosensory neurons are molecularly heterogenous, falling into at least 13 transcriptomic classes^33–35^. However, we do not yet know how many functionally different sub-types exist. Studies on the different types of sensory neurons have largely focused on those projecting to skin^36^. For the sensation of deep tissue, it is clear that Aβ neurons innervating deep tissues go beyond the proprioceptors with endings in muscle spindles and tendon organs^9–12^. Recent advances in single cell sequencing^33,37^ offer the possibility of uncovering better genetic markers for these other Aβ subtypes, including those that might mediate innocuous deep tissue sensation.

What is the molecular mechanism for pressure sensing? ΡΙΕΖΟ2 is critically required for touch discrimination, vibration sensing and proprioception^13–15^. Studies in mice and humans demonstrate that high threshold mechanical stimuli, particularly acute mechanical pain, are detected by a different mechanism^38,39^. The ability of patients with ΡΙΕΖΟ2 Deficiency Syndrome to sense innocuous pressure exposes the existence of an additional type of mechanosensation that also does not require ΡΙΕΖΟ2. Recently, several genes have been proposed to function as mechanosensitive ion channels^39–42^ and await evaluation of their in vivo roles in pressure sensation.

The current study is limited by the rarity of both Aβ-deafferentation and ΡΙΕΖΟ2 Deficiency Syndrome, leading by necessity to small sample sizes of patients and limited range of ages. It is possible that the pressure-sensing deficit observed in the Aβ-deafferented patients results in part from central changes due to many years without normal touch input. Yet, the preserved heat and pain thresholds in these patients - and the fact that healthy individuals lost deep pressure sensation after blockade of Aβ-fibers - suggests that these deficits relate more directly to their Aβ-deafferentation.

In our daily lives, we integrate information from our different sensory systems to perform basic tasks. Pressure sensation provides key information that, when absent, greatly affects quality of life^17,43,44^. While the cause of profound motor impairments in both the Aβ-deafferented and ΡΙΕΖΟ2 Deficiency Syndrome are certainly due to the loss of proprioception, it is notable that one major difference between these two syndromes is that people with ΡΙΕΖΟ2 Deficiency Syndrome retain the ability to sense pressure. Notably, the ΡΙΕΖΟ2 Deficiency Syndrome individuals are born without proprioception and develop alternative strategies throughout childhood to perform motor functions. It is tempting to speculate that, along with vision, deep tissue pressure sensation provides another key source of sensory input that these individuals use to partially compensate for their deficits.

## Methods

### Participants

This study was approved by the NIH CNS Institutional Review Board. All participants provided written informed consent to participate and all relevant ethical guidelines for human subjects research were followed. Aβ-deafferented and ΡΙΕΖΟ2 Deficiency Syndrome patients were referred to our group by clinicians and collaborators for further study. Healthy control participants were selected based on age and sex from participants in a broad screening protocol at our institute (NCCIH). Potential participants were scheduled for a telephone screening interview during which the study procedures were described and eligibility criteria were reviewed. Participants underwent medical screening for unstable medical or psychiatric conditions and any abnormalities of the skin or nerves. All participants provided informed consent and were financially compensated for their time.

### Nerve block testing in healthy volunteers

Five healthy volunteers (two females aged 21 and 25 and three males aged 21, 24, and 25) completed the nerve block portion of the study. In addition to the placement of the nerve block, these volunteers underwent repeated tests of vibration, temperature, and deep pressure perception, both before the placement of the cuff and during the nerve block procedure (i.e., while the cuff was inflated). Following the application of the cuff, the participants placed their arm on a pillow and a visual barrier was positioned to obscure their view of the blocked arm and test stimuli. They were also given noise-isolating headphones playing white noise. These volunteers did not participate as controls in any other parts of the study.

### Patient and matched healthy control testing

Two patients with a rare sensory neuronopathy syndrome causing Aβ-deafferentation (female aged 69 and male aged 65), who have been previously studied (e.g.,^19^), participated in the deep pressure discrimination, detection threshold, and sensory integration testing. Eight neurologically intact adults (4 females aged 64, 67, 69, and 69 and 4 males aged 65, 67, 68, and 72) participated as controls for the Aβ-deafferented group. Seven individuals with inherited loss-of-function mutations in the mechanically-gated ion channel ΡΙΕΖΟ2 (ΡΙΕΖΟ2 Deficiency Syndrome) also participated: three females (aged 12, 32, and 36) and four males (aged 14, 16, 19 and 43). One of these participants had a medical contraindication (deep vein thrombosis) for leg compression tasks, therefore *N* = 6 completed this task. A total of 14 healthy volunteers (8-11 per task as research spanned several years) participated as controls for the ΡΙΕΖΟ2 Deficiency Syndrome group (6 females aged 19, 19, 20, 21, 22, and 23 and 8 males aged 20, 20, 20, 21, 21, 21, 22, and 25). All individuals with ΡΙΕΖΟ2 deficiency syndrome fit the conserved clinical presentation that includes congenital hypotonia, neonatal respiratory distress, and difficulty feeding. Hip dysplasia, joint contracture, and hypermobility were common early findings. Motor development was delayed with acquisition of independent ambulation in late childhood to adolescence in 5 of the 7 patients despite absence of muscle weakness. All patients developed childhood onset progressive scoliosis.

The patients and their matched controls underwent three tests of deep pressure perception and one sensory integration test of pressure sensing with versus without visual feedback. During all deep pressure tasks the participants wore noise-isolating headphones playing white noise and had a visual barrier obscuring their vision of the stimuli.

### Statistics

Due to low sample sizes, we performed permutation t-tests to compare the performance of patients and controls on each task^45,46^. A permutation t-test is a nonparametric alternative to the Student’s t-test that tests the mean group difference against test distributions based on randomly permuted assignments of participants to groups, avoiding assumptions about the underlying data distribution. Permutation p-values were computed from 9999 random assignments of data to group. All tests were one-sided to detect loss of sensory function after nerve block or in the patient groups. All statistical analysis were performed in R using the coin package^47^.

### Nerve block procedures in healthy participants (Fig. 1a)

Each participant held their left arm above their head for ~1 min while a licensed healthcare practitioner exsanguinated the arm. Next, a blood pressure cuff (Hokanson Vascular Straight Segmental Cuff, Model SC12L^TM^, 12 × 124 cm) was wrapped around the brachium of the participant’s left arm and rapidly inflated, using an electric pump, to a pressure ~100 mmHg above the participant’s systolic blood pressure. The participant then rested their arm, dorsal side down, on a pillow in front of them. Sensory testing included the measures delineated below. Participants were instructed that we were measuring their sensory perception at baseline, then placing a nerve block, then looking at how various sensations changed over time. The cuff was released after completion of testing procedures, or if C-fiber function was lost before completion of procedures, or upon request for any reason (including pain, discomfort, anxiety, or worsening mood), or when one hour had elapsed.

#### Vibration perception during nerve block

Prior to and following the placement of the nerve block, the ability of the participants to perceive vibration was repeatedly assessed using a custom device that administered an ~200 Hz vibration to a 1.3 × 4 cm region of skin. The device was placed against the left dorsal forearm, close to the wrist, and activated for a random duration between one and six seconds. Participants responded verbally when they perceived the onset and offset of the vibration stimulus (i.e., “On” or “Off”). Vibration perception was operationalized as the percentage of onsets and offsets correctly identified. The vibration perception test was repeatedly administered until the release of the nerve block for any of the reasons listed above.

#### Heat perception thresholds during nerve block

Prior to and following the placement of the nerve block, the temperature at which the participants perceived warm stimuli was repeatedly assessed by a threshold task using a contact thermode (Medoc Pathway Model ATS 30 × 30 mm Thermal Stimulator Probes). The thermode was placed against the left ventral forearm, approximately midway between the wrist and elbow, and set to 32 °C. Heat perception thresholds were assessed by increasing the temperature of the thermode at a uniform and gradual rate (1 °C/s) until the participant indicated their perception of heat by responding with a button press. The heat perception threshold test was repeatedly administered until the release of the nerve block for any of the reasons listed above.

#### Hand pressure perception during nerve block

The ability of the participants to perceive deep pressure was assessed prior to the nerve block and again after participants showed complete (or substantial, if complete elimination was not obtained) loss of vibration perception. Controlled, oscillating deep pressure was administered to each hand for ~20 s via a commercial hand massager device (Daiwa Felicity Electric Compression Hand Massagers). Each participant rated the intensity of the massage on each hand using a visual analog scale (VAS) with anchors of “no sensation” (coded as 0) to “highest possible intensity” (coded as 100).

### Sensory testing in patients and matched healthy controls

#### Pressure intensity perception

A custom designed compression device, which allowed us to experimentally control the rate and amount of pressure, was used to inflate individual chambers of a commercial leg compression sleeve (Chattanooga Group PresSsion 8 Chamber Garments) fitted around the participant’s lower left calf (See Fig. 2b). The zone of compression was ~13 cm, beginning just above the ankle. Airflow to two chambers of the compression sleeve was supplied by compressed air tanks and regulated by an in-house custom device. The device converted USB signaling into electrical current to drive a flow regulator, and also included sleeve pressure sensing for digitizing and delivery back to USB. Sleeve inflation was controlled via Matlab^48^ programming, causing pressure to reach the target peak and then to passively drop to the target baseline.

Aβ-deafferented patients and matched healthy controls rated the intensity of two series of pressure stimuli, each containing eight trials. On each trial the sleeve oscillated five times between 10 and 30 mmHg (low series) and 15 and 65 mmHg (high series). Participants made a single rating of the perceived intensity at the end of each series on a VAS scale ranging from no sensation (coded as 0) to pain threshold (midpoint; coded as 50) to pain tolerance (coded as 100). ΡΙΕΖΟ2 Deficiency Syndrome patients and matched healthy controls completed the same task except that each series contained six trials (rather than eight) and participants made ratings of perceived intensity twice per series (after every three trials) on the same visual analog scale described above. Median intensity ratings and interquartile intervals were calculated for data representation purposes.

#### Pressure discrimination

Using the same custom compression device, we used a two-alternative forced choice discrimination task to test all participants in their ability to discriminate between different intensities of pressure. On each trial, different levels of pressure were administered sequentially, and participants indicated whether the first or second stimulus was stronger. The first block of testing included 12 randomized trials with a difference of 30 mmHg between the two stimuli (6 trials each of 30 vs 60 mmHg and 60 vs 90 mmHg). If participants performed below 75% accuracy, task difficulty was decreased to a pressure difference of 60 mmHg (30 vs 90 mmHg) and then to a difference of 90 mmHg (30 vs 120 mmHg). Several ΡΙΕΖΟ2 Deficiency Syndrome participants received fewer trials due to time constraints and superior performance on a more difficult discrimination set (differences of 15 mmHg). For each participant the percentage of correct responses was calculated.

#### Pressure thresholds

Given the Aβ-deafferented patients’ inability to perceive pressure sensations up to 120 mmHg, we performed pressure perception threshold tasks using stimulation devices that allowed the application of high pressures to determine if there was a higher level of innocuous pressure the patients could perceive, as well as to ascertain pressure pain thresholds. These tasks were repeated on two body parts with different surface areas – the lower leg and the thumb – to test whether deficits were consistent. The pressure threshold task was not administered to the ΡΙΕΖΟ2 patients since they showed no deficit in pressure discrimination.

Large Surface Area: A threshold task was used to determine what level of pressure first elicited percepts of pressure and pressure pain. The in-house device used during the discrimination task could not inflate to the pressure levels necessary for this task, so a clinical standard, manual blood pressure cuff was used instead. The blood pressure cuff (Welch Allyn FlexiPort blood pressure cuff; adult size 11: 25–34 cm circumference, 13 cm length) was wrapped around the upper calf of participants’ left and right legs and one cuff at a time was gradually inflated (~5 mmHg each second) using a manual hand pump. For trials of pressure detection, participants were instructed to verbally indicate when they perceived pressure around either leg. For trials of pain perception, they were instructed to report when they first perceived any kind of pain or “when the pressure started to hurt at all.” There were 10 trials of pressure detection and 2-4 of pressure pain perception for each participant, with an inter-stimulus interval of at least 10 s. Mean pressure levels and standard deviations were calculated for data representation purposes.

Small Surface Area: A pressure algometer (Wagner Instruments) with a blunted circular tip (1 cm diameter) was pressed manually into participants’ left thumbnail, with the thumb placed on a solid surface (see hand position in Fig. 2g). There were three trials of pressure detection and three trials of pressure pain perception for each participant. The experimenter gradually increased downward pressure on the thumbnail starting from 0 kg/cm^2^, with pressure increasing at a rate of ~0.5 kg/cm^2^ per second. Participants were instructed to indicate the moment that they felt any pressure or change in sensation (pressure threshold) or when they first perceived any kind of pain (pressure pain threshold). Mean pressure levels and standard deviations were calculated for data representation purposes.

#### Active pressure

We designed a test aimed at measuring how deep pressure perception is utilized in the specific task of applying and maintaining a constant pressure to a flat surface using one’s fingers. The participant was comfortably seated on a chair in front of a table with the volar side of the forearm of their dominant hand resting on the tabletop. Their fingers (excluding thumb) were cupped over and touching the surface of a postal scale. The digital USB postal scale M-25 (Dymo, USA) was modified so the internal load cell output was fed to PhidgetBridge (Phidget, Canada) connected to a PC laptop computer through a Universal Serial Bus connector. Custom written Python software managed data acquisition from the load cell at 62.5 Hz. Scaled data were simultaneously saved and plotted in real time on the laptop screen. The laptop was placed on the table, with the scale in front, so that the screen was directly in the participant’s line of vision. Participants were instructed to apply as much force to the scale with their fingers as needed to keep a force readout line (in black) at the same level as a guide line (in red). At the start of the test, the red guide line appeared on the screen. Two different values of force setpoint (20 g and 150 g) were tested. 20 g spread over a ~1 cm^2^ finger pad corresponds to ~14 mmHg and engages mostly cutaneous touch receptors; 150 g spread over a ~1 cm^2^ finger pad corresponds to ~110 mmHg and is predicted to activate deeper tissue receptors.

The participants were also informed that at *t* = 10 s the guide line would disappear and then re-appear at *t* = 20 s, and that their task meanwhile was to keep the applied force constant without visual feedback. Each trial lasted 30 s and trials were repeated three times per participant with force setpoints pseudo-randomized. Before data collection, all participants were allowed one test trial to familiarize themselves with the task. Recorded data were analyzed using Matlab^48^; data were smoothed over a 1 s window and trials were averaged. Performance was measured as the mean deviation from the target setpoint (MD (*t*)) at a given time point $MD\left( t \right) = \sqrt {(SP(t) - AV(t))^2}$ where SP is a setpoint value and AV actual readout value. For numerical analysis of MD(*t*) the last 5 s of the trial were averaged.

### Reporting summary

Further information on research design is available in the Nature Research Reporting Summary linked to this article.

## Supplementary information

Reporting Summary

## Data Availability

Source data are provided with this paper.

## Source data

Source Data

## Code availability

The code used to conduct the pressure tasks is available on request from the corresponding author [ATC].
