# Supplementary material for: Innocuous pressure sensation requires A-type afferents but not functional ΡΙΕΖΟ2 channels in humans
Source: Nat Commun. 2021 Jan 28;12:657. doi: 10.1038/s41467-021-20939-5 (PMC7844252; doi:10.1038/s41467-021-20939-5)
Supplement: Supplementary file 1 — Reporting Summary [file 41467_2021_20939_MOESM1_ESM.pdf]

nature research

Corresponding author(s): M. Catherine Bushnell and Alexander T. Chesler

Last updated by author(s): Dec 8, 2020

Reporting Summary

Nature Research wishes to improve the reproducibility of the work that we publish. This form provides structure for consistency and transparency in reporting. For further information on Nature Research policies, see our Editorial Policies and the Editorial Policy Checklist.

Statistics

For all statistical analyses, confirm that the following items are present in the figure legend, table legend, main text, or Methods section.

n/a	Confirmed	
☐	☒	The exact sample size (*n*) for each experimental group/condition, given as a discrete number and unit of measurement
☐	☒	A statement on whether measurements were taken from distinct samples or whether the same sample was measured repeatedly
☐	☒	The statistical test(s) used AND whether they are one- or two-sided<br>*Only common tests should be described solely by name; describe more complex techniques in the Methods section.*
☐	☒	A description of all covariates tested
☐	☒	A description of any assumptions or corrections, such as tests of normality and adjustment for multiple comparisons
☐	☒	A full description of the statistical parameters including central tendency (e.g. means) or other basic estimates (e.g. regression coefficient) AND variation (e.g. standard deviation) or associated estimates of uncertainty (e.g. confidence intervals)
☐	☒	For null hypothesis testing, the test statistic (e.g. *F*, *t*, *r*) with confidence intervals, effect sizes, degrees of freedom and *P* value noted<br>*Give P values as exact values whenever suitable.*
☒	☐	For Bayesian analysis, information on the choice of priors and Markov chain Monte Carlo settings
☒	☐	For hierarchical and complex designs, identification of the appropriate level for tests and full reporting of outcomes
☒	☐	Estimates of effect sizes (e.g. Cohen's *d*, Pearson's *r*), indicating how they were calculated

Our web collection on statistics for biologists contains articles on many of the points above.

Software and code

Policy information about availability of computer code

Data collection	Data were collected using standard procedures as outlined in the methods. Matlab 2016 was used. Custom software made in Python are available from the corresponding author upon reasonable request
Data analysis	Data were analyzed using standard statistical approaches and software (R 4.0 using the coin package) as described in the methods

For manuscripts utilizing custom algorithms or software that are central to the research but not yet described in published literature, software must be made available to editors and reviewers. We strongly encourage code deposition in a community repository (e.g. GitHub). See the Nature Research guidelines for submitting code & software for further information.

Data

Policy information about availability of data

All manuscripts must include a data availability statement. This statement should provide the following information, where applicable:

- Accession codes, unique identifiers, or web links for publicly available datasets
- A list of figures that have associated raw data
- A description of any restrictions on data availability

The datasets generated during and/or analysed during the current study are available from the corresponding author on reasonable request.

Field-specific reporting

Life sciences study design

All studies must disclose on these points even when the disclosure is negative.

Sample size	Sample size was based upon the very low number of patients diagnosed with AB deafferentation or PIEZO2-LOF. All patients available for travel to NIH in the time period of study were included in the patient samples. Control sample size was selected as N = 8 for each group after initial manuscript review deemed the number of controls too low and N = 8 would provide sufficient power for the magnitude of effects we were observing.
Data exclusions	No data were excluded except in the nerve block study if AB sensory loss did not occur as expected or if the procedure was aborted early due to pain or health considerations. Only subjects with successful nerve block and normal loss of AB sensation are described here so that the response to deep pressure can be compared to the timing of AB loss.
Replication	Findings cannot be replicated until or unless more patients with AB deafferentation or PIEZO-LOF are identified and available to research. However, the many different tests of pressure sensing conducted on each patient and control provide convergent evidence for our finding. Individual tests were not repeated on separate occasions.
Randomization	No randomization; comparisons are between patients and controls. All nerve block study procedures are within-subject. Covariates were not controlled because subjects were matched on age and sex.
Blinding	Blinding was not possible due to the obvious anatomical and motor differences in the patients. Most tasks were administered with automated devices and computer responses so as to avoid bias on the part of the experimenters.

Reporting for specific materials, systems and methods

We require information from authors about some types of materials, experimental systems and methods used in many studies. Here, indicate whether each material, system or method listed is relevant to your study. If you are not sure if a list item applies to your research, read the appropriate section before selecting a response.

Materials & experimental systems

n/a	Involved in the study	
☒	☐	Antibodies
☒	☐	Eukaryotic cell lines
☒	☐	Palaeontology and archaeology
☒	☐	Animals and other organisms
☐	☒	Human research participants
☒	☐	Clinical data
☒	☐	Dual use research of concern

Methods

n/a	Involved in the study	
☒	☐	ChIP-seq
☒	☐	Flow cytometry
☒	☐	MRI-based neuroimaging

Human research participants

Policy information about studies involving human research participants

Population characteristics	The AB-deafferented patients were 65 and 69 years old and PIEZO2-LOF patients were ages 12-36. Patient histories are described in detail in other works cited in the current paper. Control participants were age and gender matched at the group level. Approximately half of both each patient and control group was female vs male. Healthy volunteers for the nerve block study were ages 21-25 and recruited through a broad phenotyping protocol at our institute.
Recruitment	All patients were recruited from physician-researcher colleagues known to be studying the AB-deafferented or PIEZO2 LOF patients. All controls were recruited from our broad sensory phenotyping protocol of healthy volunteers and were selected by age and gender.
Ethics oversight	The NIH CNS IRB approved this research.

Note that full information on the approval of the study protocol must also be provided in the manuscript.
